# Decoding MTSS1: Turning Genetic Clues into Cardiac Cures

**DOI:** 10.1016/j.jacbts.2025.101412

**Published:** 2025-11-24

**Authors:** Julien Ochala

**Affiliations:** Department of Biomedical Sciences, University of Copenhagen, Copenhagen, Denmark; Myocardial Homeostasis and Cardiac Injury Program, Centro Nacional de Investigaciones Cardiovasculares, Madrid, Spain

**Keywords:** dilated cardiomyopathy, heart failure, sarcomere, TTN

**Figure:**
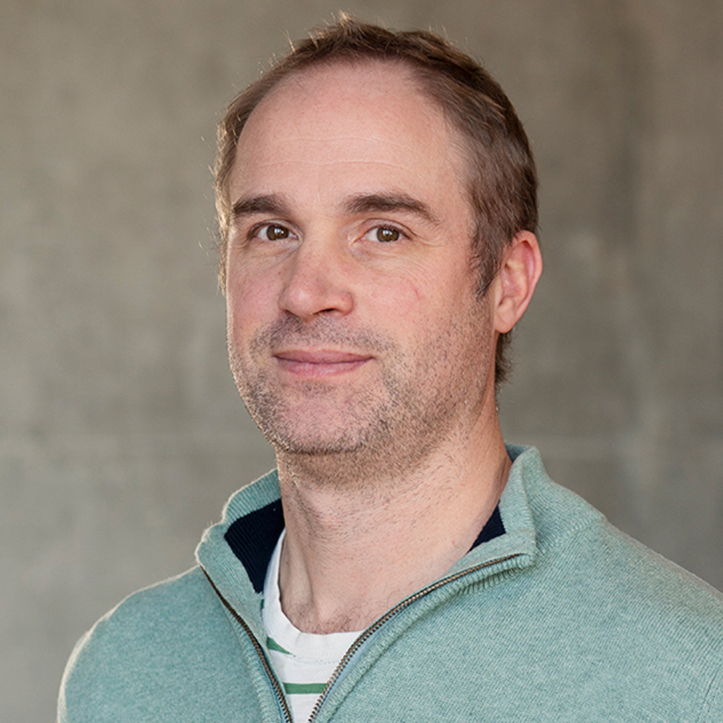

Dilated cardiomyopathy (DCM) is a structural myocardial disorder notably defined by progressive ventricular remodeling and reduced ejection fraction, leading to heart failure. Its prevalence ranges between 1:250 and 1:2,500, with genetic variants accounting for approximately 30% to 50% of cases.^1^^,^^2^ Despite clinical guidelines relieving and managing symptoms, approved treatments directly targeting the molecular mechanisms remain scarce. The study by Kleppe et al^3^ in this issue of *JACC: Basic to Translational Science* delivers a profound advance by combining human genetics and sophisticated cellular models to identify MTSS1 as a key modulator of sarcomere biology and contractility. This elegant work exemplifies the integration of genetic discovery and mechanistic biology to reveal promising therapeutic avenues for DCM.

Building on genome-wide association studies that link variants reducing cardiac MTSS1 expression with improved contractility and better symptoms outcomes,^4^^,^^5^ the authors identify the key role of this protein (MTSS1) in DCM patients carrying truncating variants in TTN. Indeed, their analysis of the UK Biobank cohort reveals that patients harboring both pathogenic TTN variants and MTSS1 expression-lowering alleles have markedly improved survival rates. This compelling human genetic evidence made the authors run further necessary mechanistic explorations. Employing induced pluripotent stem cell-derived cardiomyocytes with variants in TTN and other DCM genes such as CSRP3 and RBM20, Kleppe et al^3^ demonstrate that siRNA-induced MTSS1 knockdown increases sarcomere numbers and enhances contractile force. High-content confocal imaging paired with machine learning delineate dose-dependent improvements in sarcomere architecture and contractility with MTSS1 suppression. In parallel, proteomic assays confirm MTSS1 link to MYO18A and related sarcomere assembly proteins, suggesting mechanisms that may directly regulate sarcomere turnover or formation. Additionally, transcriptional profiling indicates up-regulated expression of key sarcomere proteins and down-regulation of heart failure markers, consolidating the functional benefits observed.

The impact of these findings extends well beyond this specific molecular target. The study by Kleppe et al^3^ strongly demonstrates how leveraging human genetic variants can guide the identification of novel therapeutic targets with clinical relevance, overcoming some limitations of traditional preclinical models vulnerable to species differences. It sets a new standard for precision cardiology, where genetic modifiers inform drug development and patient stratification, ultimately aiming to deliver tailored therapies for genetically defined heart failure populations.

Looking forward, this work opens up several transformative avenues. First, it strengthens the idea of focusing on sarcomere quantity and quality to treat heart failure. Second, the methodological framework established here, integrating large-scale human genetics, high-throughput phenotypic assays, and advanced machine learning, presents a replicable blueprint for accelerating drug target discovery in cardiovascular disease. This convergence of disciplines promises increased efficiency and precision in identifying, validating, and prioritizing molecular targets for clinical translation. Third, patient stratification based on genetic modifiers, exemplified by MTSS1 expression levels, heralds an era of personalized cardiovascular medicine. Therapies targeting MTSS1 may be most effective in individuals harboring specific pathogenic mutations, emphasizing the need to incorporate genomic data into future clinical trial designs to optimize efficacy and reduce variability. Finally, the translational path toward therapy demands development of safe, cardiac-specific delivery systems for MTSS1 modulators, whether siRNA-based agents or small molecule inhibitors. Longitudinal studies assessing durability, off-target effects, and immune responses will be critical. The high unmet need in monogenic DCM and the absence of causative treatments provide impetus for rapid advancement of these strategies through preclinical and early clinical phases.

In summary, the comprehensive approach by Kleppe et al^3^ in this issue of *JACC: Basic to Translational Science* connects human genetic insights with cellular mechanisms to position MTSS1 as a modifiable regulator of sarcomere biology and contractility in genetic DCM. This breakthrough strengthens the therapeutic paradigm that takes into consideration variants and restores sarcomere integrity in the context of heart failure.

## Funding Support and Author Disclosures

This work was generously funded by a Novo Nordisk Foundation project grant (NNF23-OC0085045) to Dr Ochala. The author has reported that she has no relationships relevant to the contents of this paper to disclose.
